# Community Core Evolution in Mobile Social Networks

**DOI:** 10.1155/2013/781281

**Published:** 2013-09-17

**Authors:** Hao Xu, Weidong Xiao, Daquan Tang, Jiuyang Tang, Zhenwen Wang

**Affiliations:** Key Laboratory for Information System Technology, National University of Defense Technology, Changsha 410073, China

## Abstract

Community detection in social networks attracts a lot of attention in the recent years. Existing methods always depict the relationship of two nodes using the temporary connection. However, these temporary connections cannot be fully recognized as the real relationships when the history connections among nodes are considered. For example, a casual visit in Facebook cannot be seen as an establishment of friendship. Hence, our question is the following: how to cluster the real friends in mobile social networks? In this paper, we study the problem of detecting the stable community core in mobile social networks. The cumulative stable contact is proposed to depict the relationship among nodes. The whole process is divided into timestamps. Nodes and their connections can be added or removed at each timestamp, and historical contacts are considered when detecting the community core. Also, community cores can be tracked through the incremental computing, which can help to recognize the evolving of community structure. Empirical studies on real-world social networks demonstrate that our proposed method can effectively detect stable community cores in mobile social networks.

## 1. Introduction

In the recent years, the way of communication among people has experienced a dramatic change. Thanks to the development of mobile communication technology, the relative geographical topology among passengers can be caught easily. Hence, clustering people in such mobile social networks, which can be further used in information recommendation and other social services, attracts more and more concerns.

There are a lot of literatures concerned with the community detection in social networks, including static approach and dynamic approach. Nodes are usually depicted as people in the real world, and links are always denoted to be the contacts among nodes. The static approach focuses on high aggregation of nodes which have the same features [1, 2]. While the dynamic approach divides the network's evolving process into lots of timestamps, they not only pay attention to clustering nodes in the network but are also concerned with the computational complexity at each timestamp [3, 4]. At each timestamp, the computational complexity depends on change of links, rather than all links in the network. It is very important when analyzing evolution of the network structure, especially with multiple timestamps. However, few of these methods consider the stability of communities between two timestamps. Intuitively, in our real world, the relationship among people will not change sharply. That is to say, for a giving link  *l*_*a*,*b*_(*t*)  which denotes a link between nodes  *a*  and  *b*  at time *t*, *l*_*a*,*b*_(*t*) cannot depict the relationship between nodes  *a*  and  *b*  exactly. The study in [5] considers the stability in community detection, but it tries to obtain a community partition under the stable modularity, rather than the stable contact.

Moreover, [6] pointed out that intercontact time among people follows the power-law distribution, which means the following: (1) we spend most of our time contacting with the “community” people; (2) there are still a lot of temporary contacts taking place between “strangers.” If all of the links are considered when detecting community, some *temporary links* among “strangers” will influence the effect. In order to eliminate the negative influence, only *familiar links* should be concerned. And our question is the following: how to find the real friends having those *familiar links* with each other in a mobile social network?

The biggest feature of mobile social network is that nodes and links are always changing. The studies in [3, 7] have classified all of the situations that occur at each timestamp into several events, including node add/remove and link (edge) add/remove. And their experiment results demonstrate that discretization of the continuous time is a useful way to model the evolution of the network. In this paper, the discretization of the continuous time is still adopted when modeling the evolution process. However, the prominent difference of our method compared with others is the discrimination between *familiar link* and *temporary link*.

Based on previous works [8], the number of *familiar links* is higher than that of *temporary links*. In other words, people who come from the same community have higher contact frequency than those who come from different communities. And the changing frequency of *familiar link* is lower than that of *temporary link*. That is to say, people who come from the same community always keep the relatively stable contact, while contacts among people who come from different communities seem to be uncertain.

Based on the discussion above, we use “community core” to solve our problem. Unlike our definition, the concept denoted by [5] is based on the nondeterministic community detection algorithm. Generally speaking, a community core in mobile social networks is the subset of a community. For links in the same community core, few changes will occur between consecutive timestamps.

The study in [9] extracts the core structure of social networks using (*α*, *β*) community. The authors have discovered the core structure by lots of overlapping (*α*, *β*) communities. However, the proposed static heuristic algorithm did not consider the dynamic features of social networks. The study in [5] analyzed the community core in evolving networks. By recursive computation, stable community cores are detected and well tracked. However, the instability of communities in [5] is caused by the instability of the nondeterministic detecting algorithm, which does not suit the community core detection under the instability of links at each timestamp.

In this paper, we propose a novel approach for community core detection in mobile social networks. The main idea of our approach is to find a partition based on stable links in a giving network. To the best of our knowledge, we are the first to find the relatively stable community core using the history cumulative contact in mobile social networks and the first to find the power-law distribution of these contacts' changing between consecutive timestamps. In order to recognize the change of community core at each timestamp, the tracking mechanism is also concerned.

The rest of this paper is organized as follows. We introduce the preliminaries used in this paper in Section 2. In Section 3, we discuss the characteristics of cumulative stable contact. Then, we present our community core detection and tracking algorithm separately in Section 4. We evaluate our algorithms in Section 5, and we finally conclude the work with Section 6.

## 2. Preliminary

In this section, we present the notion and the mobile network model that we will use throughout the paper.

**Definition 1 (mobile social network)**

A mobile social network is denoted as  *G* = 〈*E*, *V*〉, where *V* is the vertex set and  *E*⊆*V* × *V*  is the link set.

The nodes and links of a mobile social network will change according to different timestamps. A mobile social network at  *T* = *t*  is denoted as  *G*(*t*) = 〈*E*(*t*), *V*(*t*)〉. Here,  *V*(*t*)  is the set of nodes that appears at  *T* = *t*, and  *E*(*t*)  is the set of links that appears at the same timestamp:  *V*(*t*)⊆*V*, and  *E*(*t*)⊆*E*.

Topologies of a certain mobile social network are always changing due to the time variation, which is the most difference compared with static networks. Like previous works, we treat the continuous time as a sequence of timestamps. Furthermore, nodes and links may be different from the consecutive timestamps. Hence, we use the following four events to describe the evolution of network: node add, node remove, link add, and link remove.

**Definition 2 (cumulative stable contact, (CSC))**

There is a CSC between two nodes  *v*_*i*_  and  *v*_*j*_  if and only if their history contact duration is higher than a threshold (we will discuss this threshold in the following section).

As mentioned before, the *temporary link* cannot depict the relationship between two nodes in the mobile social network. Inversely, two nodes that disconnect at *T* = *t* cannot demonstrate that they are irrelevant. Considering the history connection among nodes, we use cumulative contact to judge the stability of links.

**Definition 3 (community core set)**

It is denoted as CRS = {CO_0_, CO_1_,…, CO_*m*_}, where CO_*i*_ is a community core and is a subset of *G* : ⋃_*i*=0_CO_*i*_(*t*)⊆*G*(*t*).

The community core at *T* = *t* is a partition about the given network, the same as the concept “community” in previous works. A community set is defined as a partition of a given network: CS = {*C*_0_, *C*_1_,…, *C*_*m*_}. However, we only focus on detecting the “useful links” rather than carry on a cluster process. Hence, some of the nodes and links may not be included in CRS(*t*) even if they appear at *T* = *t*. Therefore, the biggest difference between community and community core is ⋃_*i*=0_CO_*i*_(*t*)⊆⋃_*j*=0_ *C*_*j*_(*t*).

## 3. Cumulative Stable Link

In this section, we study the characteristics of CSC. First, a well-known mobile social network is introduced. Then, a stable link extraction method is proposed to find the CSC. Finally, we discuss the distribution of the *change of CSC* (CCSC).

### 3.1. Dataset

Due to the increasing concern about mobile social networks, various datasets about people's behavior are collected by researchers. The studies in [8, 10] have collected the traces information about attendants of INFOCOM06 and SIGCOMM09 separately. The features of these datasets are as follows. (1) These datasets include not only the contact information but also the attributes of attendants. The SIGCOMM09 has 76 attendants, and the INFOCOM06 has 78 attendants. (2) Both of these datasets contain several days traces information; more than 300000 timestamps can be used to describe the evolution of networks.

SIGCOMM09 collects the traces information among attendants in SIGCOMM 2009. The dataset not only records the contact time of each device pair but also includes the profile of each attendant such as country, city, institute, and interest. The contact information is recorded in the form of <*timestamp, user_id, seen_user_id,…*>, which means the scanning time, the scanning device, and the discovered device. Hence, the cumulative contact pair at each timestamp is easier to obtain.

Similar to SIGCOMM09, INFOCOM06 collects the contact traces among attendants in INFOCOM 2006. Each participant was asked to fill in a questionnaire that included name, nationality, affiliation, country, and other items. The contact information is also well refined by the author so that in the form of <*user_id, seen_(user_id), start time, end time,…*>, which means scanning device, discovered device and their duration contact time.

### 3.2. Stable Link Extraction

Both the SIGCOMM09 and INFOCOM06 contain connection duration between each pair of nodes. We use a contact matrix **M** denoting the contact among nodes.  *m*_*i*,*j*_(*t*) is the cumulative contact duration between *v*_*i*_  and  *v*_*j*_  from *T* = 0 to *T* = *t*.

The study in [8] has studied the correlation between regularity and familiarity on Cambridge students, and it is observed that most of contacts among nodes reveal a short duration, while few of them have long duration, which is denoted as “community”. In this paper, we use **M**′ = [*m*_*i*,*j*_′] to denote whether *v*_*i*_ and *v*_*j*_ have a contact duration higher than a threshold *δ* · max⁡(*m*_*i*,*j*_). Then, we cluster the attendants into several groups by their friendship graphs which are extracted from the two datasets as follows:

(1) $$\begin{array}{c} m_{ij}^{\prime }=\{\begin{array}{cc} m_{ij}, & m_{ij}\geq \delta \cdot \max\left(m\right),\\ 0, & m_{ij}<\delta \cdot \max\left(m\right). \end{array} \end{array}$$

### 3.3. Distribution of CCSC

We first construct a contact duration matrix **M** = [*m*_*i*,*j*_], where  *m*_*i*,*j*_  presents the history contact duration between  *v*_*i*_  and  *v*_*j*_  during the whole lifetime of the network (INFOCOM06:  *T* = [6207, 340927]; SIGCOMM09:  *T* = [21, 349811]). Without loss of generality, we denote  **X**  and  **Y**  as two consecutive  **M** and then compute the *change of history contact *(CHC), which is depicted as the distance of  **X**  and  **Y**  using

(2) $$\begin{array}{c} \text{Distance}\left(X,Y\right)=\underset{j}{\sum }\underset{i}{\sum }s\left(i,j\right),\\ s\left(i,j\right)=\{\begin{array}{cc} 0, & X_{ij}=Y_{ij},\\ 1, & X_{ij}\neq Y_{ij}. \end{array} \end{array}$$

**Figure 1:**
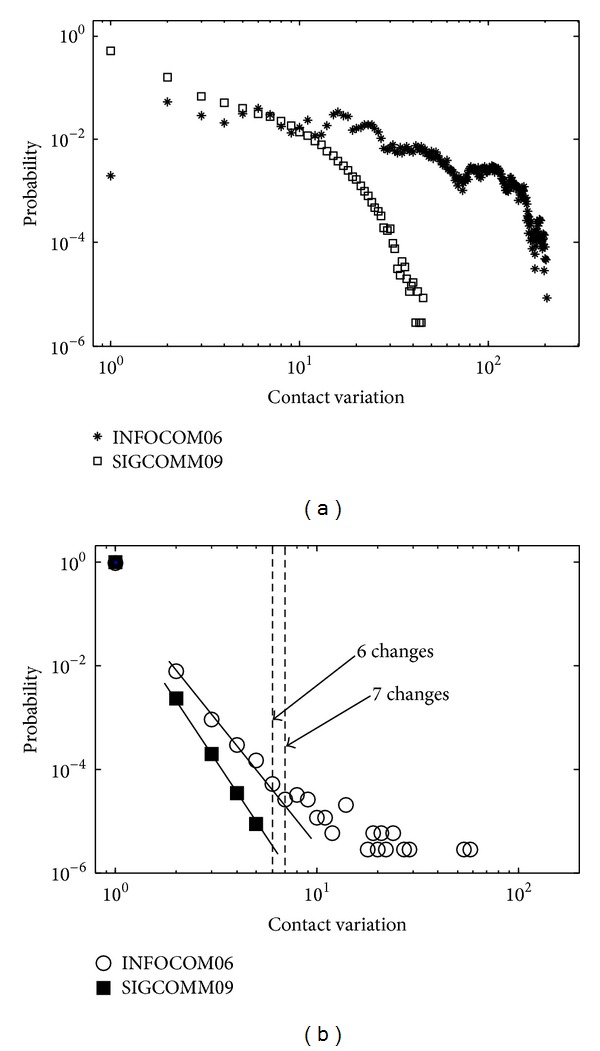
(a) Distribution of history contact duration. (b) Distribution of CCSC.

The distribution of the distance is plotted in the log-log scale (Figure 1). The power-law distribution of intercontact time in the mobile social network is fully discussed in the existing literatures. However, the change of contacts in consecutive timestamps does not follow the power-law distribution. Then, we use **M**′, where *m*_*i*,*j*_′ denotes whether a link between  *v*_*i*_  and  *v*_*j*_  is a CSC or not, and we compute the distance of two consecutive **M**′; then, the CCSC at different timestamps is obtained (Figure 1(a)). The distribution of CCSC is plotted in Figure 1(b) using log-log scale. It is clear that the CCSC extracted from SIGCOMM09 follows the power-law distribution, ranging from 2 changes to 6 changes. In INFOCOM06, when the changes range from 2 to 7, the CCSC also follows the power-law distribution. The diversity of distribution between history contact duration and change of cumulative stable contact might be caused by removal of the temporary contact. Considering two people in the real world, the more familiar, the more stable their relationship is. Moreover, people denoted as “familiar” have longer contact duration and contact time, which has been proven in previous works. According to the discussion above, the CSC removes the temporary links among nodes.

## 4. Community Core Evolution

In this section, we first introduce the community core detection algorithm and then discuss the community core tracking mechanism in mobile social networks.

### 4.1. Community Core Detection

**Figure 2:**
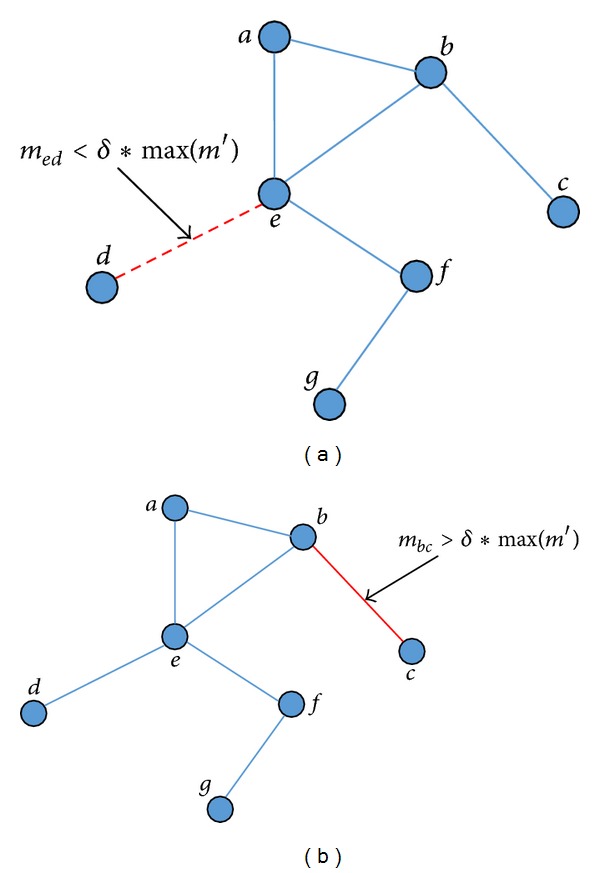
Two events cause the structure variation of network. (a) A link between nodes  *b*  and  *c*  is added into the network because *m*_*b*,*c*_′ > *δ*∗max⁡(*m*′). (b) A link between nodes  *d*  and  *e*  is removed from the network because *m*_*d*,*e*_′ < *δ*∗max⁡(*m*′).

Let us first discuss the topology change of the network, which is constantly updated by nodes and links changing through different timestamps. The increasing nodes or links can be decomposed as a sequence of node or link insertions, while the decreasing nodes or links can be decomposed as a sequence of node or link removals. We define four events that may cause the evolution of network: node add, node remove, link add, and link remove. However, in the definition above, the community core is based on links between two nodes. Hence, a single node without associated CSC cannot exist in the community core. Then, we refine the events as shown in Figure 2.

*Link Add*. The cumulative contact between *v*_*i*_ and *v*_*j*_ is higher than the current threshold; then link *e*_*i*,*j*_ that is associated with two nodes *v*_*i*_ and *v*_*j*_ is added to a community core CO. Both *v*_*i*_ and *v*_*j*_ will be added to CO, even if any of them did not belong to CO.

*Link Remove.* The cumulative contact between *v*_*i*_ and *v*_*j*_ is lower than the current threshold; then link *e*_*i*,*j*_ that is associated with two nodes *v*_*i*_ and *v*_*j*_ is removed from a community core CO. If *v*_*i*_ or *v*_*j*_ has no link associated with other nodes in CO, then the corresponding node will be removed from CO.

**Figure 3:**
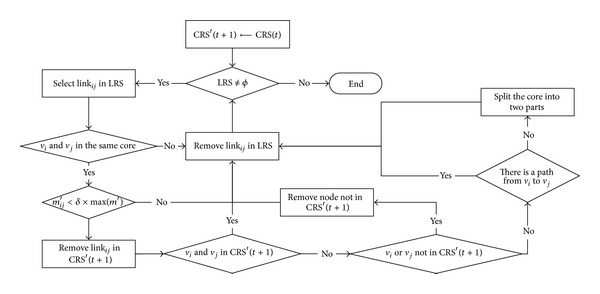
Basic operation procedure of link remove.

**Figure 4:**
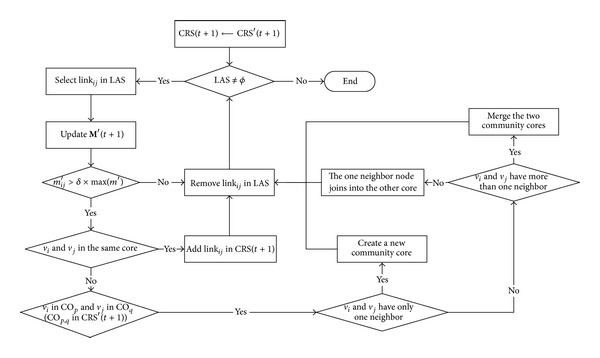
Basic operation procedure of link add.

The basic operation procedure of community core detection at one timestamp is described in Figures 3 and 4. We extract the decreasing and increasing links at each timestamp and then use LRS and LAS to denote the decreasing link set and the increasing link set separately.

Giving a certain community core partition, the *Link Remove* will result in deleting existing community cores (Figure 3), while *Link Add* will result in expanding the community core or inserting a new community core (Figure 4). The procedure can be divided into two phases. Firstly, we treat the Link Remove set and refine the existing community core through the existence of connected path. If there is no path connected, the link will be removed from CO. Secondly, by updating the max contact duration, addition link and its nodes will be added into the existing community core or a new community core.

### 4.2. Evolution of Community Core

In order to study the evolution process of community core, we should track the community core at each timestamp. How to distinguish two community cores in the consecutive timestamps is the biggest problem about tracking. 

#### 4.2.1. Model

**Figure 5:**
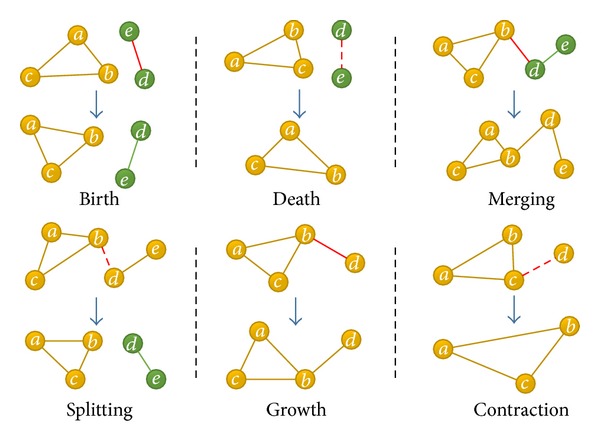
Six events provide variation of community cores. The red solid/dash line denotes the link add/remove separately, and different colors mean different community cores.

According to Definition 3, the evolution of community core can be presented by CRS_0_, CRS_1_,…, CRS_*k*_,…. For a community core at *T* = *t*, CRS(*t*) = {CO_0_, CO_1_,…, CO_*m*_}, and *L*(*t*) = {*l*_0_, *l*_1_,…, *l*_*n*_} is denoted as the label set of community cores identifying a community core at *T* = *t* uniquely. In the previous literatures, there can be seen a broad consensus on the basic events that can be used to describe the evolution of dynamic communities [3, 7, 11]. We extend and specify these events as shown in Figure 5.

*Birth*. It is the emergence of a new community core at *T* = *t* + 1, and there is no corresponding community core in CRS(*t*). A community core CO_new_ which is labeled as *l*_new_ is created in CRS(*t* + 1) at *T* = *t* + 1, while CO_new_ ∉ CRS(*t*). That is, for any *l*_new_ ∈ *L*(*t* + 1), there is *l*_new_ ∉ *L*(*t*). 

*Death*. It is the disappearance of a community core CO_old_ at *T* = *t* + 1 as CO_old_ exists in CRS(*t*) at *T* = *t*. There is no corresponding community core in CRS(*t* + 1). If we use *l*_old_ denoting core label of CO_old_, then the death of a community core CO_old_ means  *l*_old_ ∈ *L*(*t*), and, for any  *l*_*s*_ ∈ *L*(*t* + 1), *l*_*s*_ ≠ *l*_old_.

*Merging*. It means that two community cores merge into one community core at *T* = *t* + 1. 

*Splitting*. A community core is divided into two separate community cores. 

*Growth*. A node joins into a community core. And this will result in no changes between *L*(*t*) and *L*(*t* + 1).

*Contraction*. A node moves out of a community core. This also will result in no changes between *L*(*t*) and *L*(*t* + 1). 

#### 4.2.2. Tracking Community Cores

In the context of the model described above, the most important thing is to identify the changes of community cores. Three questions should be answered. (1) Where does the core come from? (2) What happened to community core after updating? (3) Where is the core going?

The study in [12] is concerned with tracking communities based on detected communities, using the Jaccard coefficient. Differing from this method, we track these cores by enhancing our algorithm. When the algorithm runs, the tracking process works simultaneously, which is triggered by the variation of links.

According to our algorithm, there exist only contact frequency between two core nodes lower than *δ* · max⁡(*m*_*i*,*j*_), and there is no other path connecting these two nodes in the original core; the splitting process will be triggered. Hence,  *v*_*i*_  and  *v*_*j*_  have their isolate core sets separately. We use node ID as the core label when creating a new community core. Initially, each node will be tagged a community label as itself. The benefits are as follows. Firstly, the finite namespace of core label will not cause muddle of naming. Secondly, community core can be tracked easier when a community core is created again. The tracking algorithm uses node ID as initial community core ID if the node is extracted to a community core, and when a node is removed from community core set, the node ID is restored.

Basic operation procedure of tracking is given as follows.

*Switch* (*events*)

 Case *Birth* (*v*_*i*_ and *v*_*j*_ form a new core):
  (3) $$\begin{array}{c} L\left(t+1\right)⟵l_{\text{new}},\;\;\;l_{\text{new}}=i\;\text{or}\;j; \end{array}$$
 Case *Death* (CO_*i*_ ∈ CRS(*t*) but CO_*i*_ ∉ CRS(*t* + 1)):
   Every node in CO_*i*_ labeled as its node ID.
 Case *Merging* (CO_*i*_ and CO_*j*_ merge into on core):
   Nodes with fewer core members change their IDs to the other core.
 Case *Splitting* (*v*_*i*_ and *v*_*j*_ have no path to connect):
   Delete *l*_old_ in *L*(*t* + 1), and *L*(*t* + 1) ← *i*, *j*, *v*_*i*_ and *v*_*j*_ change label as their node ID.
 Case *Growth* (*v*_*i*_ join into an existing community core):
   
*v*_*i*_ changes label as the community core.
 Case *Contraction* (*v*_*i*_ moves out of community core):
   
*v*_*i*_ changes label as its node ID.

## 5. Evaluation

In this section, we discuss the evolution of community core, which is detected and tracked by our algorithm. COPRA [13] is a well-known efficient algorithm of fast community detection, and we choose COPRA to compare with our algorithm.

### 5.1. Contact Variation

**Figure 6:**
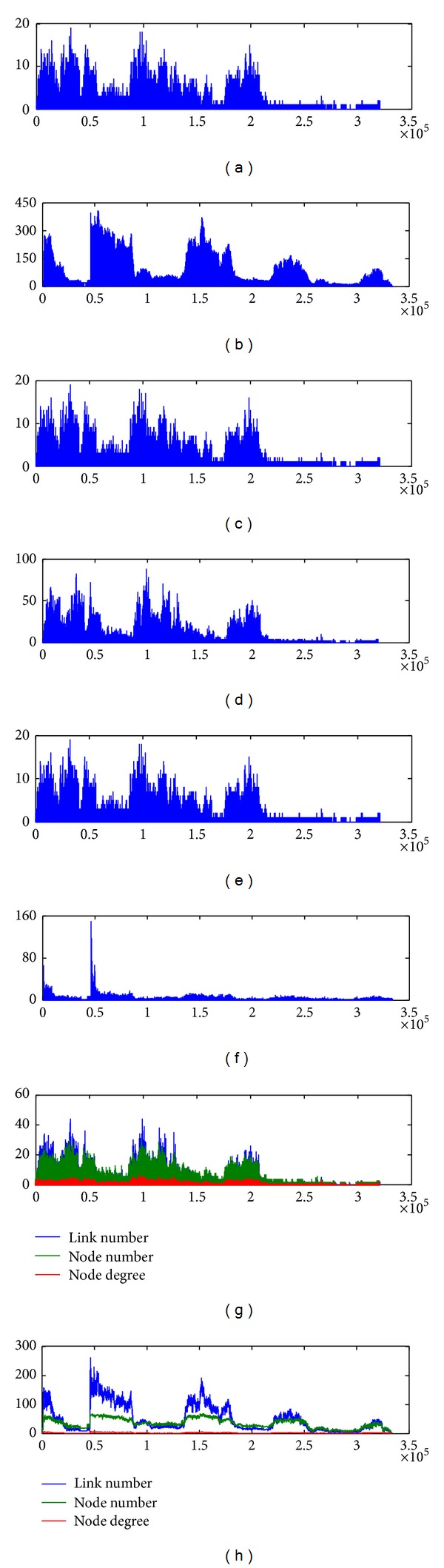
Description of two datasets. SIGCOMM09 records the discrete contact time among nodes, while INFOCOM06 records the continuous contact time. (a) Total link number (SIGCOMM09), (b) total link number (INFOCOM06), (c) link add (SIGCOMM09), (d) link add (INFOCOM06), (e) link remove (SIGCOMM09), (f) link remove (INFOCOM06), (g) network feature (SIGCOMM09), and (h) network feature (INFOCOM06).

In order to reveal the community core evolution briefly, we first show the contact variation of both SIGCOMM09 and INFOCOM06 under the whole lifetime (Figure 6). Due to the difference of data preprocessing, SIGCOMM09 records the discrete contact time among nodes, while INFOCOM06 records the continuous contact time. According to the description of SIGCOMM09, each device performs a periodic Bluetooth device discovery every 120 ± 10.24 seconds (randomized) for 10.24 seconds. When a device finds others, it records the current time. However, this mechanism can only record one contact time in a period for the same pair, which will miss some contact information. Hence, the total link change, link add, and link remove of SIGCOMM09 almost have no differences.

### 5.2. Change of 0-1 Contact Matrix

**Figure 7:**
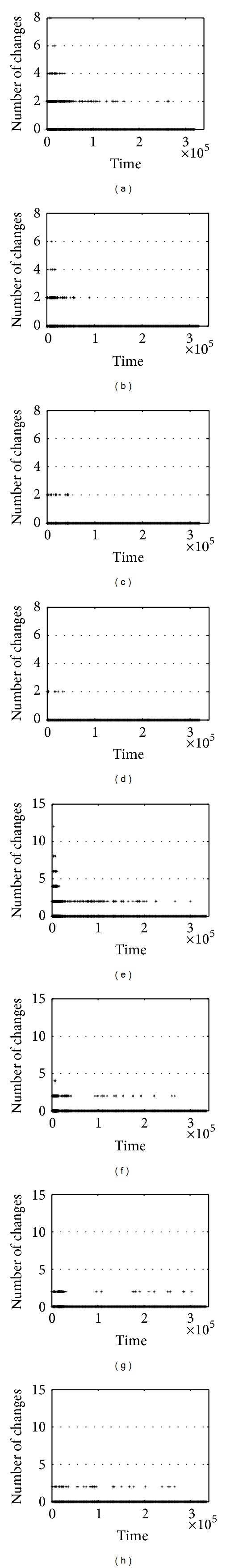
Number of changes in 0-1 matrix between two consecutive timestamps. Above: number of changes in SIGCOMM09 under *δ* = 0.2, 0.4, 0.6, and 0.8 (from (a) to (d)). Below: number of changes in INFOCOM06 under *δ* = 0.2, 0.4, 0.6, and 0.8 (from (e) to (h)).

According to **M**′, the 0-1 contact matrix **B** = [*b*_*i*,*j*_] can be obtained. **M**′ changes when the time increases, and the maximum contact duration among nodes may be changed, which results in the variation of **B**. The change of **B** during the whole collection procedure is plotted in Figure 7. Consider the following:

(4) $$\begin{array}{c} b_{ij}=\{\begin{array}{cc} 1, & {m^{\prime }}_{ij}\geq \delta \cdot \max\left(m^{\prime }\right),\\ 0, & {m^{\prime }}_{ij}<\delta \cdot \max\left(m^{\prime }\right). \end{array} \end{array}$$

As shown in Figure 7, most of the changes occur at the beginning of collection, and they are then gradually diminished over time. The number of changes reduces by the increasing of *δ*. Meanwhile, in SIGCOMM09, the maximum changes of 0-1 contact matrix under *δ* = 0.2, 0.4, 0.6, 0.8 are 8, 6, 4, and 2, respectively, while in INFOCOM06, the values are 12, 4, 2, and 2. With *δ* increasing, the maximum changes of 0-1 contact matrix are reduced.

### 5.3. Selected Node Count

One of the biggest differences between community and community core is the number of clustered nodes. In other words, the community core of a mobile social network is the subset of the whole community. According to previous works in [8], few of nodes can be classified as “familiar strangers” and “friends,” and nodes in the “community” are even less. Hence, only the node pairs which have high contact frequency can be selected to the community core.

**Figure 8:**
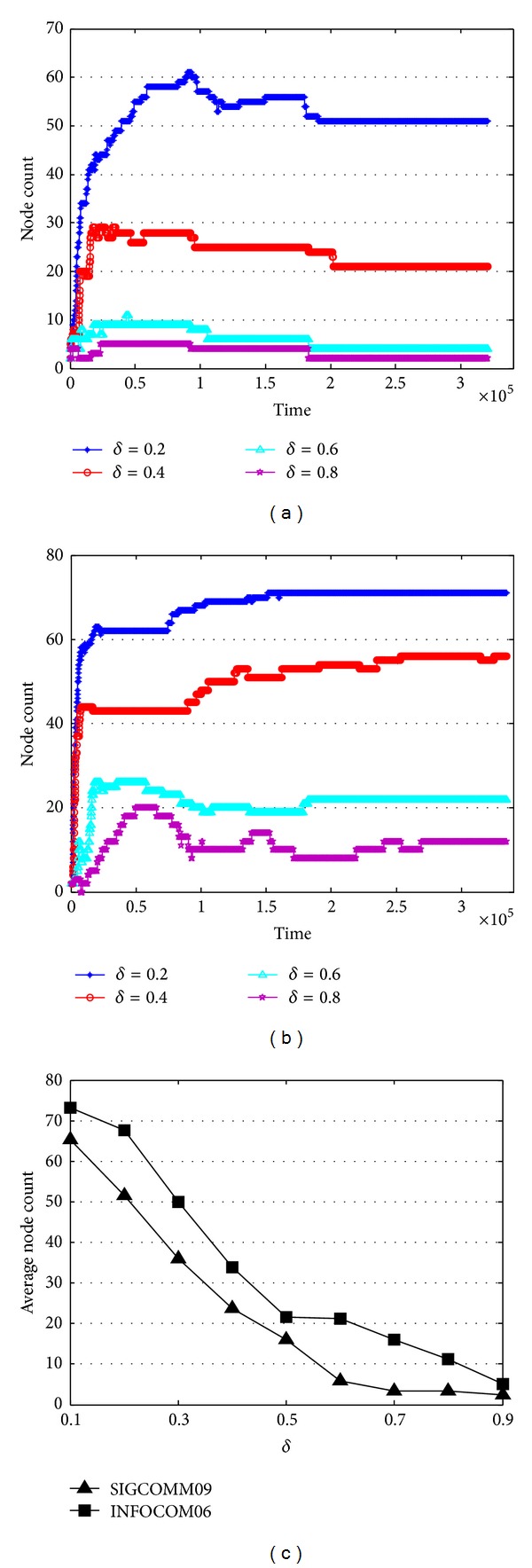
Number of nodes in community core. (a) Variation in number of nodes in community core in SIGCOMM09. (b) Variation in number of nodes in community core in INFOCOM06. (c) Average number of nodes in community core in the two datasets under different *δ*.

Intuitively, improving *δ* will result in fewer selected contacts and nodes, which is illustrated in Figures 8(a) and 8(b). Let us first consider SIGCOMM09, when time goes by, the selected nodes become more and more stable. In the first day of data collection, the selected node changes dramatically, especially under the low *δ*. Then, the stable duration of selected nodes prolonged, and the selected nodes no longer have change after about 2∗10^5^ seconds. Compared with different *δ*, the lower *δ* will result in a higher selected nodes, which is meaningless for community cores (when *δ* = 0.2, during the data collection, the maximum selected nodes in SIGCOMM09 are 61, and the maximum selected nodes in INFOCOM06 are 71), while the higher the *δ* is, the fewer nodes are selected to construct the community core. A brief comparison of average selected nodes under different *δ* is depicted in Figure 8(c). The same as discussed above, the average of selected nodes decreases when *δ* increases. Although the selected nodes increase with the decreasing of *δ*, the variation of selected nodes in INFOCOM06 still has little difference. Unlike SIGCOMM09 the selected nodes in INFOCOM06 change frequently, even at the end of the data collection. This phenomenon can reflect the fact that in SIGCOMM09, the “friends” of attendants are relatively stable, and participants usually contact with familiar people, while in INFOCOM06, contacts among strangers are more frequent than in SIGCOMM09; hence, the selected node changes more often. Nevertheless, the variation of selected nodes in SIGCOMM09 and INFOCOM06 is relatively stable after 2∗10^5^ seconds, which is important according to features of community core.

**Figure 9:**
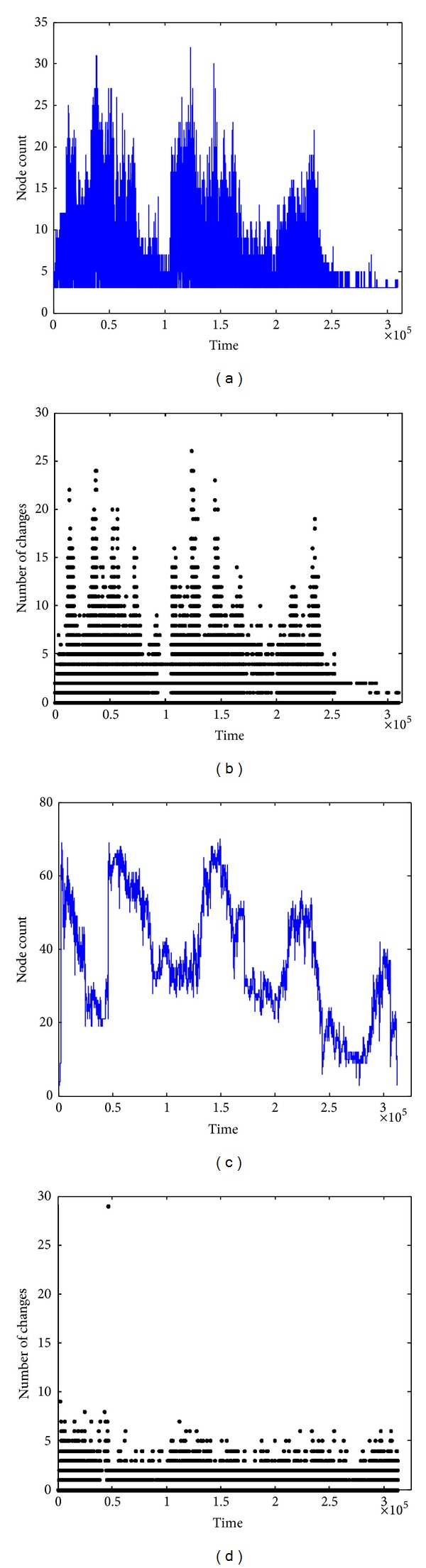
Number of nodes in community detected by COPRA. (a) Node number at each timestamp in communities (SIGCOMM09). (b) Number of node changes in community between two consecutive timestamps (SIGCOMM09). (c) Node number at each timestamp in communities (SIGCOMM09). (d) Number of node changes in community between two consecutive timestamps (INFOCOM06).

The number of nodes in communities detected by COPRA is depicted in Figure 9. Different from our algorithm, the number of nodes detected by COPRA is highly unstable. And the number of changes between two consecutive timestamps is also very large compared with our algorithm.

### 5.4. Number of Community Cores

**Figure 10:**
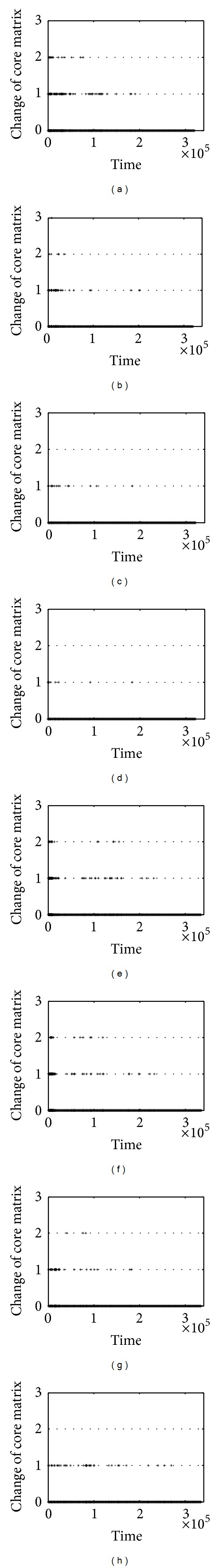
Number of changes in core matrix between two consecutive timestamps. Above: number of changes in SIGCOMM09 under *δ* = 0.2, 0.4, 0.6, and 0.8 (from (a) to (d)). Below: number of changes in INFOCOM06 under *δ* = 0.2, 0.4, 0.6, and 0.8 (from (e) to (h)).

A community core set is composed of selected nodes, which divides a mobile social network into pieces of closely connected fragments. Hence, the community core number in a community set determines the fragmentation degree of this mobile social network. After running our algorithm, the community core count in each timestamp under different *δ* is obtained (Figure 10).

**Figure 11:**
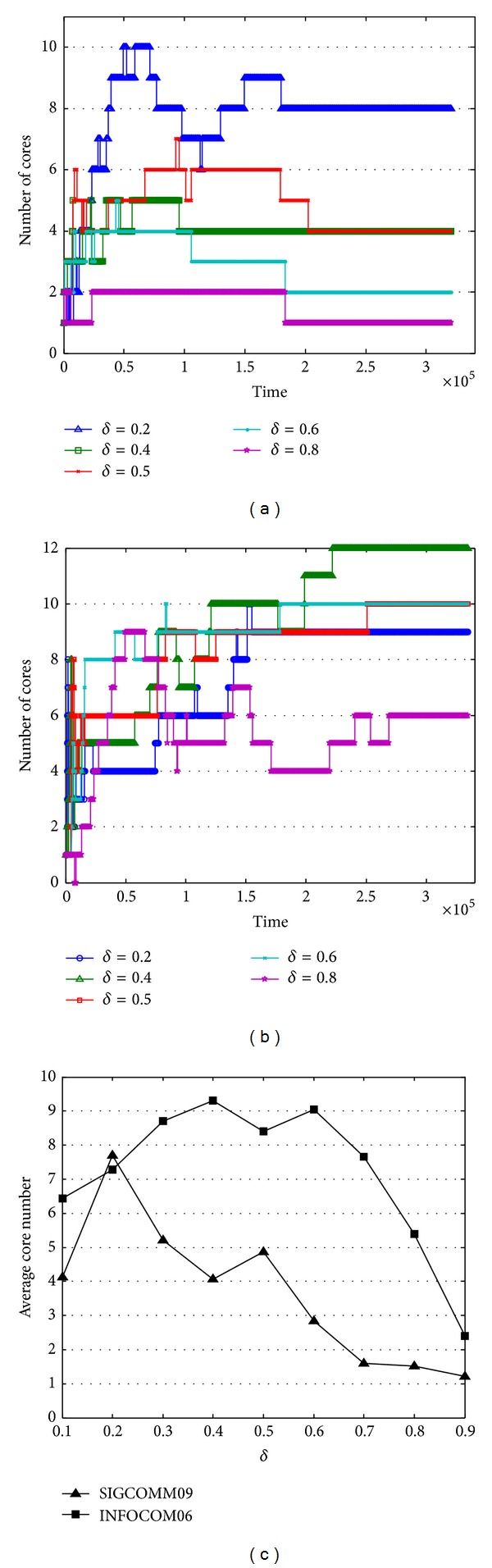
Number of community cores. (a) Variation in number of community core in SIGCOMM09. (b) Variation in number of community cores in INFOCOM06. (c) Average number of community cores in the two datasets under different *δ*.

As depicted in Figure 11(a), after about 2∗10^5^ seconds, the community count under each different *δ* becomes fixed, which is the same as the selected nodes. However, the core number in INFOCOM06 is more complicated. Like the variation of selected node, the number of community core fluctuates with time changes; see Figure 11(b).

As mentioned before, this is due to the frequent contact among strangers. In other words, attendants in SGICOMM09 have a more fixed social circle than in INFOCOM06. The last but the most important feature of community core count is that the core number is not monotonically changing with changes. We can see in Figure 11(c) that the maximum average core count (*≈*7.71) in SIGCOMM09 appears when *δ* = 0.2. After a reduction to *δ* = 0.4, the average core count rises approximate to 4.87 when *δ* = 5. The same phenomenon appears in INFOCOM06: the core count reaches the max value at *δ* = 4 (*≈*9.31). Then, it declines approximate to 8.4 at *δ* = 5 and grows approximate to 9.05 at *δ* = 6. The reason for this situation can be explained by two sides. On one hand, the higher will filter out more contacts, which makes more fragments of the mobile network and increases the number of community cores. On the other hand, some network fragments with low contact frequency will be moved out of the community core set entirely, which results in the reduction of cores. Hence, the number of cores fluctuates under the different. 

**Figure 12:**
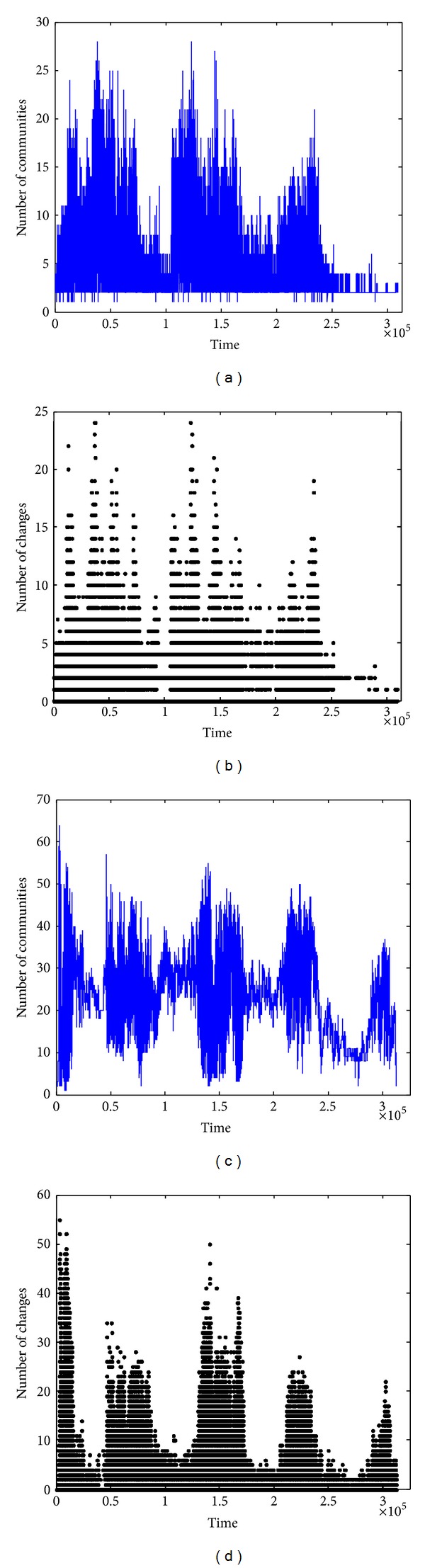
Number of communities detected by COPRA. (a) Number of communities at each timestamp (SIGCOMM09). (b) Number of community changes between two consecutive timestamps (SIGCOMM09). (c) Number of communities at each timestamp (INFOCOM06). (d) Number of community changes between two consecutive timestamps (INFOCOM06).

Stability is very important to the community core. Compared with Figures 11 and 12, the community core is much more stable than traditional community detection algorithm. The number of communities detected by COPRA is depicted in Figure 12, which reveals high instability. And the number of changes between two consecutive timestamps is also very large compared with our algorithm.

### 5.5. Change of Core Matrix

In order to evaluate the change of community core set between consecutive timestamps, we use the distance function discussed in Section 3.3 to illustrate the difference between consecutive community core sets. 

The distance function requires that the two matrices have the same line and row number. However, the nodes and links in the community core set between consecutive timestamps are different. According to the tracking algorithm, nodes in the mobile social networks are assigned an initial community core label which is equal to their node ID. And the finite namespace of core label ensures that the label will not be beyond the scope of node ID. Then, we construct a community core matrix **C** **M** = [*cm*_*i*,*j*_], where

(5) $$\begin{array}{c} {cm}_{ij}=\{\begin{array}{cc} \text{node}\;\text{ID}, & i,j\notin \text{CRS},\\ \text{core}\;\text{ID}, & i,j\in \text{CRS}. \end{array} \end{array}$$

Note that although node ID is considered in computation, it will not influence the final result of distance.

**Figure 13:**
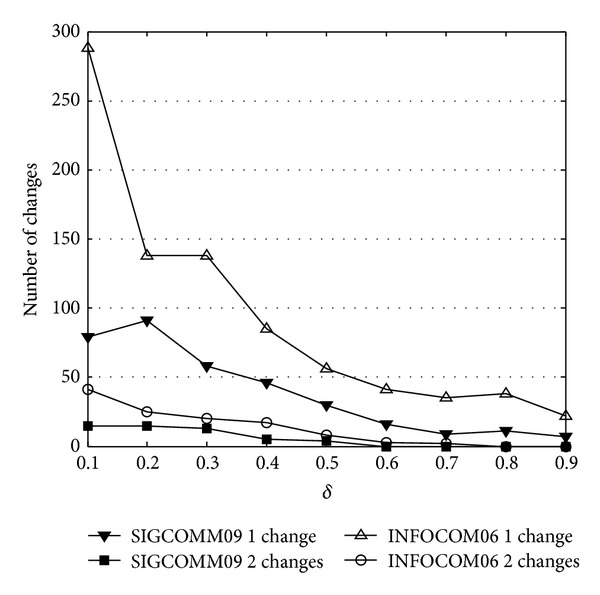
Number of changes in core matrix with different *δ*.

The distance under different *δ* is depicted in Figure 10. It can be observed that the majority of consecutive core matrices have no changes. Only few of them have 1 different element, and the variation of 2 elements is even less (Figure 13). With *δ* increasing, the variation of core matrix reduces accordingly. This illustrates that the higher the *δ* is, the more sensitive the community core becomes. Considering the variation time, most of the changes occur at the beginning of data collection, which is also consistent with the change of selected nodes and the core number.

### 5.6. Community Core Tracking

**Figure 14:**
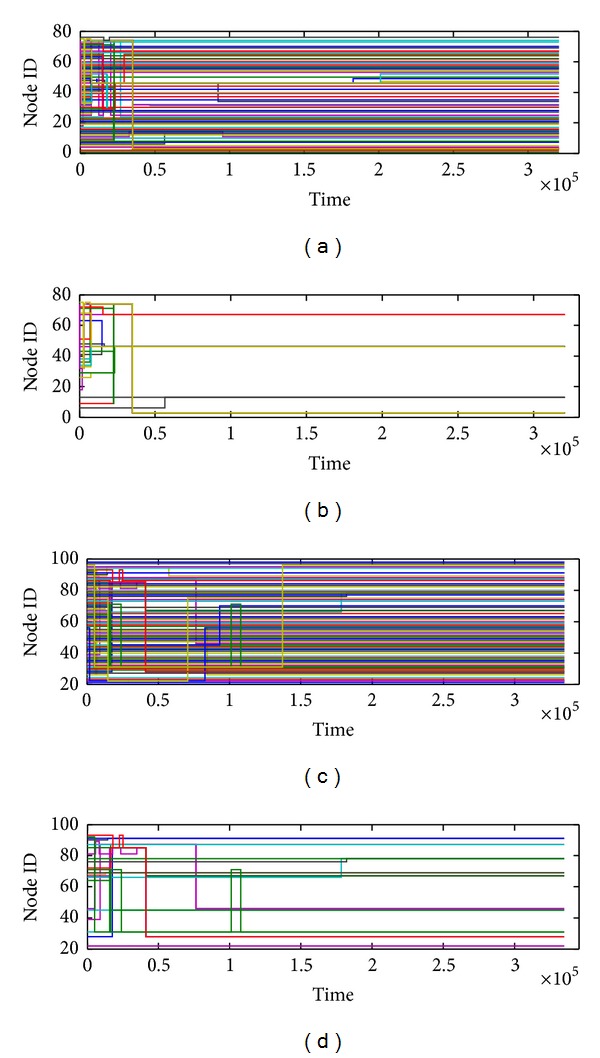
(a) Original community core evolution in SIGCOMM09 with *δ* = 0.4. (b) Refined community core evolution in SIGCOMM09 with *δ* = 0.4. (c) Original community core evolution in INFOCOM06 with *δ* = 0.6. (d) Refined community core evolution in INFOCOM06 with *δ* = 0.6.

In this part, we focus on the visualization of community core evolution. First, we extract the core ID of each node from community core matrix. If a node does not belong to any cores, it is labeled as its node ID. Then, we get the community core at each timestamp. Finally, the ID including only one node is removed. The community core set extracted from the two datasets is presented in Figure 14. According to the selected node and the core number under the different *δ*, we choose *δ* = 0.4 in SIGCOMM09 and *δ* = 0.6 in INFOCOM06 to display the evolution of community cores.

Figures 14(a) and 14(c) include nodes in the community cores and the noncore nodes. If a node does not belong to any community cores, its ID will be a straight line. Besides, if a node is selected as the community core, the ID will change to the core ID. Figures 14(b) and 14(d) are the refined core traces, including nodes belonging to the community cores only. Compared with Figure 11, the evolution of community cores appears directly.

## 6. Conclusions

In this paper, we mainly analyze the community core in mobile social networks. Firstly, the change of cumulative stable contact is discussed. And then, we propose the community core detection algorithm to extract the community core from two experimental mobile social networks. Finally, we introduce a label-based community core tracking algorithm, which can briefly display the evolution of community core. Compared with traditional community detection algorithms, we show that the community core extracted by our algorithm is stable and that it can be further used in network analysis in mobile social networks.
